# Between Amyloids and Aggregation Lies a Connection with Strength and Adhesion

**DOI:** 10.1155/2014/815102

**Published:** 2014-02-02

**Authors:** Peter N. Lipke, Caleen Ramsook, Melissa C. Garcia-Sherman, Desmond N. Jackson, Cho X. J. Chan, Michael Bois, Stephen A. Klotz

**Affiliations:** 1 Biology Department, Brooklyn College, The City University of New York, 2900 Bedford Avenue, Brooklyn, NY 11210, USA; 2 The Graduate Center, The City University of New York, New York, NY 10016, USA; 3 Section of Infectious Diseases, University of Arizona, Tucson, AZ 85724, USA

## Abstract

We tell of a journey that led to discovery of amyloids formed by yeast cell adhesins and their importance in biofilms and host immunity. We begin with the identification of the adhesin functional amyloid-forming sequences that mediate fiber formation *in vitro*. Atomic force microscopy and confocal microscopy show 2-dimensional amyloid “nanodomains” on the surface of cells that are activated for adhesion. These nanodomains are arrays of adhesin molecules that bind multivalent ligands with high avidity. Nanodomains form when adhesin molecules are stretched in the AFM or under laminar flow. Treatment with antiamyloid perturbants or mutation of the amyloid sequence prevents adhesion nanodomain formation and activation. We are now discovering biological consequences. Adhesin nanodomains promote formation and maintenance of biofilms, which are microbial communities. Also, in abscesses within candidiasis patients, we find adhesin amyloids on the surface of the fungi. In both human infection and a *Caenorhabditis elegans* infection model, the presence of fungal surface amyloids elicits anti-inflammatory responses. Thus, this is a story of how fungal adhesins respond to extension forces through formation of cell surface amyloid nanodomains, with key consequences for biofilm formation and host responses.

## 1. Introduction

We have discovered functional amyloids that cluster cell adhesion molecules on the surface of fungal cells. This clustering increases the strength of cell-to-cell binding by increasing the number of bonds formed between cells at a given moment. In this outlook, we will recap what we know and how we discovered it and then explore some of the consequences and potential applications of the discovery.

This is a story of a scientific discovery, with many approaches initiated at a series of lunches. Klotz and Lipke connected scientifically when we shared a table at lunch one day at the American Society for Microbiology meeting in 2004. Gaur and Klotz had cloned and expressed a gene (*ALA1*, now named *ALS5*) for a *C. albicans* cell adhesion protein [1]. They had named it *α*-agglutinin-like adhesin for a *Saccharomyces cerevisiae* homolog. (Serendipitously, it was the Lipke lab along with collaborator Janet Kurjan that had characterized and sequenced *α*-agglutinin; Lipke and Kurjan [2]). A discussion about how to find the ligands of Als5p led to a long and fruitful collaboration and to our first joint papers published in that same year.

## 2. What Is Apparent from the Als Sequence and Activity

**Figure 1:**
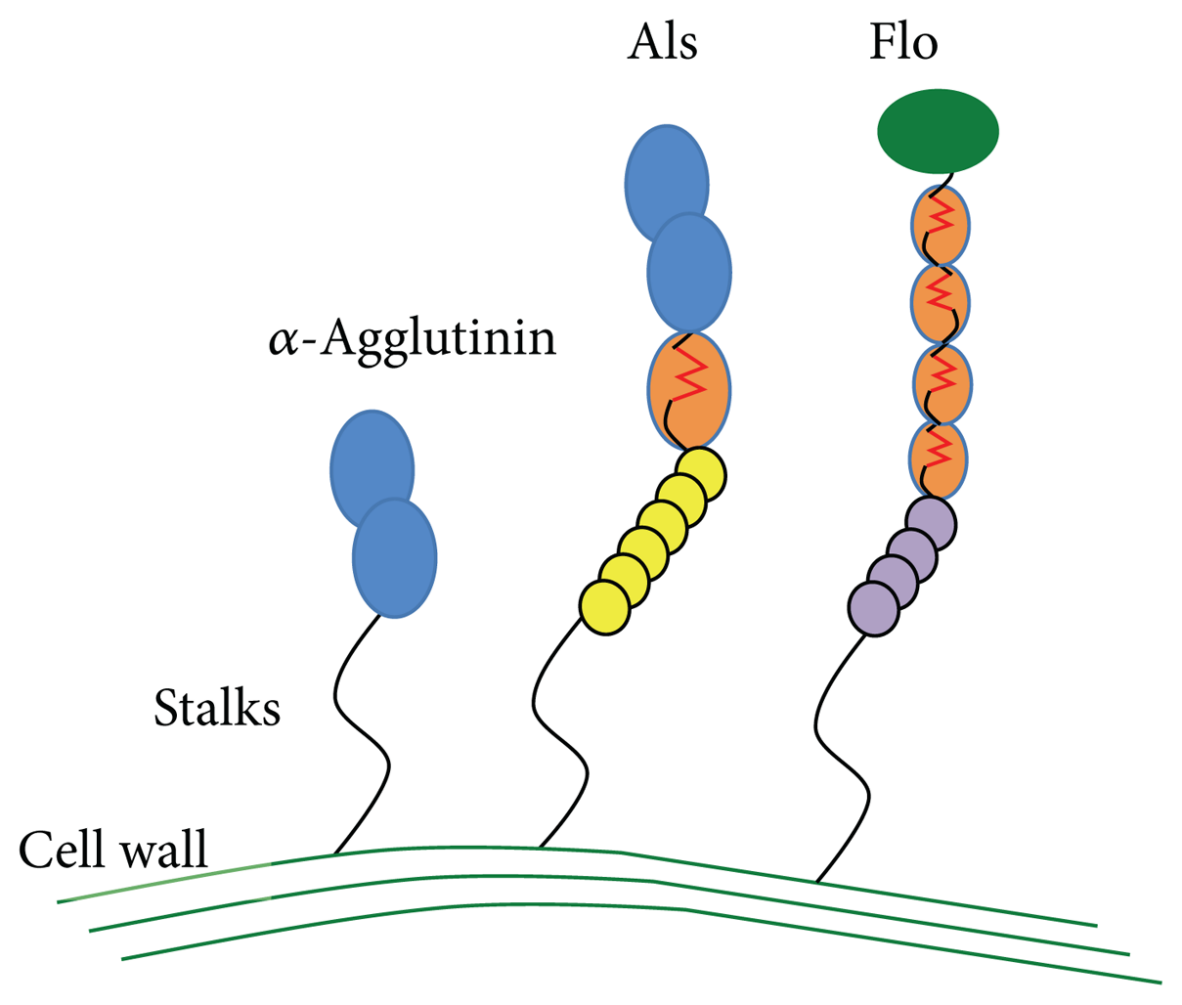
Models of fungal adhesins. The N-terminals are at the top, and the C-terminals at the bottom are covalently bound to cell wall glucans. Various domains are cartooned, including Ig-like invasin domains (blue), a green lectin domain, amyloid-forming domains (orange with red amyloid sequences), and tandem repeats in orange, yellow, and purple.

**Figure 2:**
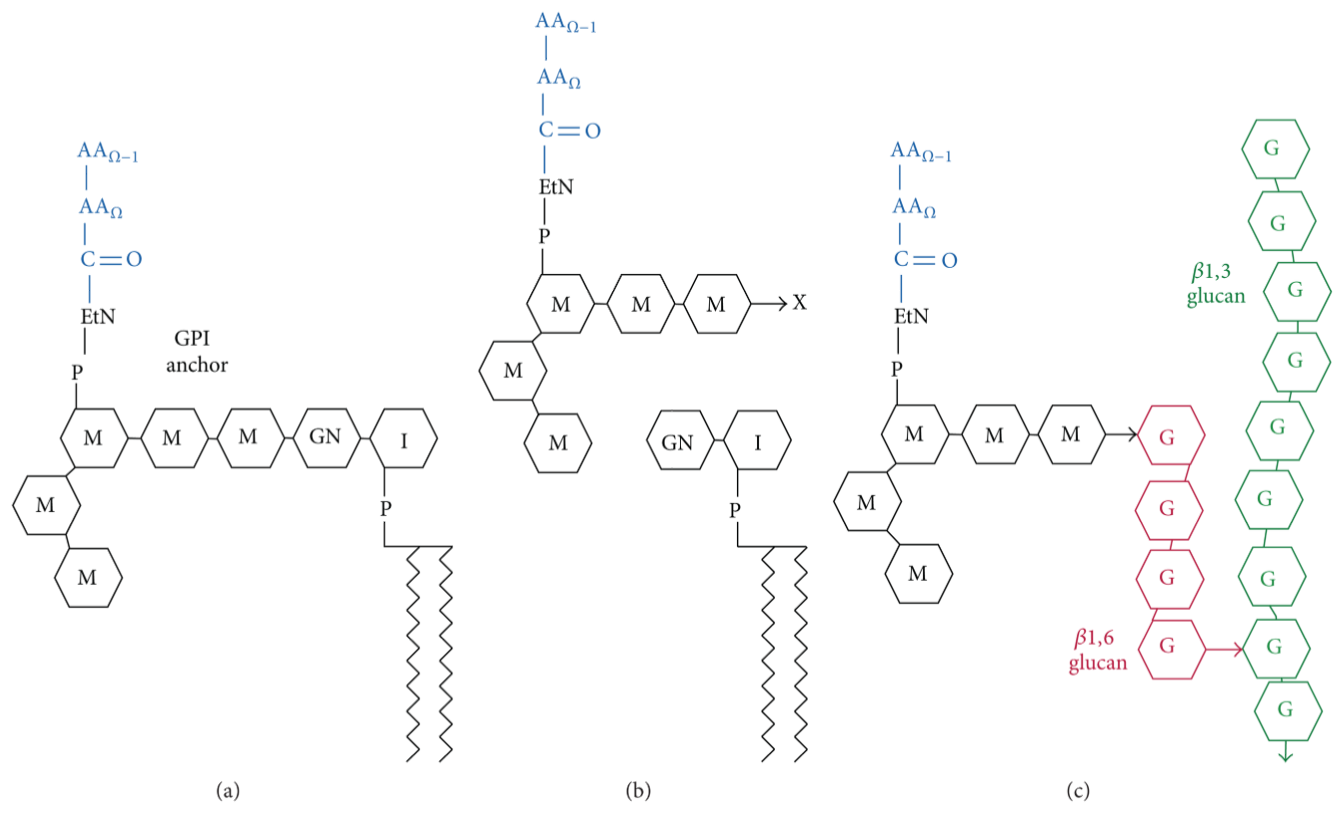
Model for transglycosylation of a GPI-anchored protein to form a covalent bond with cell wall glucan. The polypeptide chain is blue, the GPI black, and the cell wall glucans red and green. Reprinted with permission from Lipke and Ovalle [28].

Previously, Hoyer et al. had identified and sequenced *ALS1* (*α*-agglutinin-like sequence), another member of a gene family that turned out to have 8 members inmost *C. albicans* strains [1, 3]. The *ALS* genes are paralogous, meaning that they are homologous sequences within one organism. The Als proteins and *α*-agglutinin are also homologous, so they share domain architecture, as cartooned in Figure 1. The encoded proteins have N-terminal secretion signal sequences followed by a pair of domains with structure and sequence motifs like those in immunoglobulins. These Ig-fold domains bind to many different peptide sequences, either as soluble peptides or as unstructured parts of proteins [4–6]. The structures of these domains have now been determined [7]. Following the Ig-like domains is a Thr-rich domain of 108 amino acids, called the T domain. This domain contains an amyloid-forming sequence in each paralog and has the most highly conserved sequence in Als proteins. C-terminal to the T region is a series of 36-amino acid tandem repeats (TR region), and various alleles and paralogs have repeat numbers ranging from 3 to 36 [8]. The repeats mediate hydrophobic effect nonspecific binding to many kinds of surfaces [9]. C-terminal to the TR region is a 300–1000 amino acid segment rich in N-glycosylation sites as well as the potential O-glycosylation sites, Ser, and Thr. By analogy with *α*-agglutinin, this region forms an extended unstructured stalk that is highly N- and O-glycosylated [10]. The stalk turns out to be highly important for clustering and amyloid formation. At the most C-terminal region is a second signal sequence, this one for addition of a glycosyl phosphatidyl inositol (GPI) anchor. This is a C-terminal addition to many cell adhesion molecules and other surface proteins in eukaryotes. In taxa other than fungi, the lipid moiety of the GPI is inserted into the plasma membrane to anchor the protein in the bilayer. In yeast, the GPI anchor is also originally membrane-associated. However, when it is attached to an adhesin, the glycan of the GPI is cleaved, and the sugar remnant still attached to the protein is transglycosylated onto cell wall glucan, thus making a covalent link between the C-terminal of the protein and the polysaccharide of the cell wall (Figure 2; [10–12]). This covalent cell wall linkage has been directly demonstrated for Als proteins as well as *α*-agglutinin [13].

Gaur and Klotz had developed a convenient aggregation assay. Magnetic beads coated with an Als5p ligand (usually fibronectin or denatured BSA) were mixed with a 100-fold excess of cells [1]. The cells used could be *C. albicans* itself, in which case the adhesion and aggregation could be attributed to any of the dozens of *C. albicans* adhesins. However, they usually used *S. cerevisiae* cells that had been engineered to express Als5p from a plasmid. The *S. cerevisiae* cells expressed, glycosylated, exported, and anchored the adhesin to the wall in a manner homologous to *C. albicans*. Because laboratory strains of *S. cerevisiae* do not express other adhesins, it is a great surface display model: any adhesive or aggregative can be attributed to the exogenous *C. albicans* adhesin. This same approach allowed others to clone and characterize other *C. albicans* adhesins [14–16]. It is this assay that we have exploited to deduce the structural and functional properties of the Als adhesins.

## 3. Fidelity of *S. cerevisiae α*-Agglutinin and Promiscuity of *C. albicans* Als Proteins

Despite their homology, there are behavioral and structural differences between *α*-agglutinin and the Als proteins. Behaviorally, the two proteins could not be more different. So far, *α*-agglutinin has one known ligand on earth: the Ag**a**2 glycopeptide subunit of its partner adhesin **a**-agglutinin, expressed on the opposite mating type [2, 17, 18]. Thus, the sexual agglutinins of *S. cerevisiae* are the most monogamous of all microbial adhesins and show specificity characteristics like the sperm-egg adhesion proteins [19, 20]. This highly specific binding is tight, with an extremely slow dissociation rate apparently limited by conformational shifting in the proteins to increase the strength of the interaction [21, 22]. In contrast, Als adhesins are promiscuous; they bind to almost anything. Our initial screen showed that Als5p and Als1p bound to 1–2 percent of peptides in a random sequence library [4]. Most of these ligands had a common sequence motif, “*τφ*+,” consisting sequentially of an amino acid common in turns, a bulky hydrophobic amino acid, and a cationic amino acid. Such sequences are extremely common in proteins, so almost all proteins are potential ligands. In addition, the sugar *α*-fucose is a ligand, and the proteins also mediate nonspecific binding to hydrophobic surfaces [9, 23, 24]. Binding of these ligands is weak, and dissociation is rapid. Therefore, Als proteins and *α*-agglutinin show opposing trends in ligand binding: broad versus specific, weak versus strong, and slow versus fast dissociation. We attributed these differences to obvious differences in structure, namely, the presence of T and TR regions in the Als adhesins but not in *α*-agglutinin (Figure 1).

## 4. Cell Surface Activation of Als Adhesins

**Figure 3:**
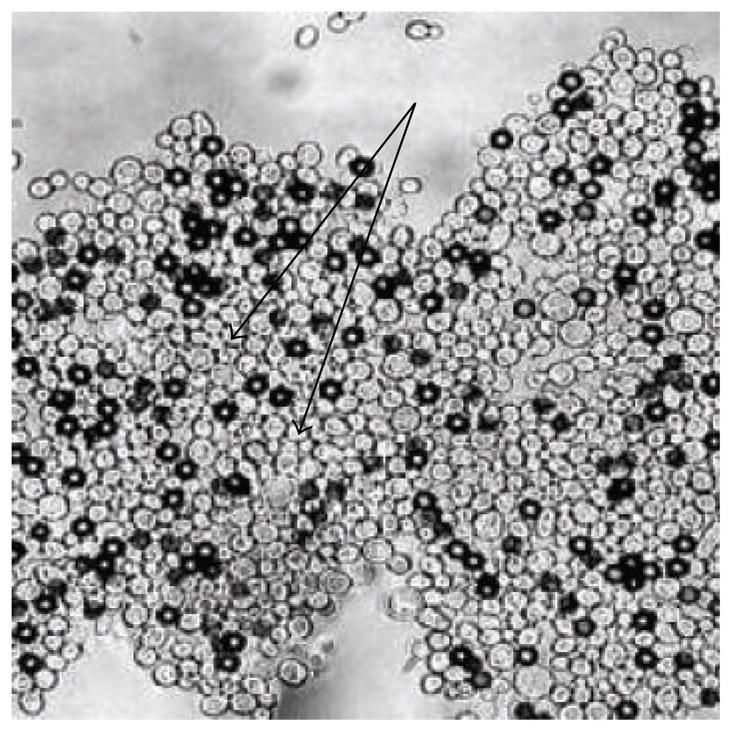
An aggregate of *S. cerevisiae* cells expressing Als5p with magnetic beads coated with fibronectin (dark spheres). Note the cell-cell aggregation in areas without beads (arrows). Modified with permission from Rauceo et al. [25].

We observed another emergent property of Als5p that although cells expressing this adhesin did not aggregate during growth, when we mixed them with ligand-coated magnetic beads, the cells became uniformly sticky. Not only did they bind to the beads, but within a few minutes the far sides of the cells became adhesive as well and formed aggregates with other cells that were not bound to beads (Figure 3; [25, 26]). Thus, the entire surface of each cell became activated for adhesion. How was this achieved? In Lipke’s lab, graduate student Jason Rauceo was interested in the problem and invited the boss out to lunch so the discussion would not be interrupted by phone calls or e-mails. Rauceo et al. tested various metabolic and signal transduction inhibitors, and none was able to block adhesion, even with the protein synthesis inhibitor cycloheximide or the metabolic poison NaN_3_ [25]. Gaur narrowed our thinking considerably by pointing out that adhesion occurs just as well in heat-killed cells. These results imply that the elements required for adhesion must exist on the cell surface before aggregation starts, and no new gene products are needed. How could this happen? One mechanism that might explain our observation is conformational changes in the adhesin proteins that lead to initiation of binding. Indeed, Rauceo et al. found that compounds that perturb protein secondary structure were effective inhibitors of cell adhesion. Among these effective inhibitors was the amyloid-binding dye Congo red [25]. Moreover, activation of the cell adhesion was accompanied by changes in surface protein structure and hydrophobicity. So even with dead cells, Als proteins underwent a conformational change accompanied by surface protein clustering, and this activated state spread around the entire surface of the cells. This property was reminiscent of the self-propagation of amyloids and prions [27].

## 5. Protein Amyloids

Amyloids are commonly known as pathological protein aggregates in chronic central nervous system diseases like Alzheimer’s and variant Creutzfeldt-Jakob (mad cow) diseases, as well as in serum amyloidosis, in which aggregates can damage the kidney [29]. However, this is a one-sided view: amyloids are actually a specific type of protein aggregate and can form from many different proteins. In amyloids, a small segment of the sequence of a protein associates with the identical sequence in other protein molecules, and the proteins assemble in fibers composed of *β*-strands aligned at right angles to the fiber axis, a structure called “cross-*β*.” Such structured aggregates are among the natural states of proteins and so are not necessarily pathological. In fact, the amyloid state is naturally rare, perhaps because proteins have evolved to avoid amyloid formation by eliminating amyloid-forming parts of the sequence or burying them inside of stable domains, where they cannot interact with amyloid sequences in other protein molecules [30–32]. Nevertheless, proteins do form amyloids *in vivo*; some are pathological, some may be neutral, and some amyloids are formed by design, the so-called functional amyloids.

## 6. A New Functional Amyloid

There is a growing number of functional amyloids, and they include assemblages of bacterial adhesins and appendages and hydrophobic protein coats on some fungi. In mammals, there are amyloids in melanosomes that template melanin synthesis and deposition. In addition, the proteins in exocrine condensed bodies consist of amyloid-like assemblies [27]. Fungal adhesin amyloids add more members to this list.

There are now a variety of approaches to predict whether a given sequence can be assembled into amyloid structures. We first heard about TANGO at a FASEB conference on protein aggregation in 2007. TANGO is a web-hosted protein structure secondary prediction based on entropy estimates for immobilizing amino acid side chain in cross-*β*-sheet aggregates such as amyloids [33]. Surprisingly, TANGO predicted a single amyloid-forming sequence in the highly conserved T domain of Als5. Within binding region of Als5p, this sequence shows 93–95%potential to form aggregated *β*-sheet structures (a value >5% is considered characteristic of amyloid formation) [33, 34]. This sequence Gly-Ile-Val-Ile-Val-Ala-Thr-Thr (GIVIVATT), is unusual among known amyloid sequences in its high content of *β*-branched aliphatic side chains and an absence of aromatic residues or Asn or Gln. Sequence gazing showed the identical sequence in the Als1p and Als3p, the most highly expressed of the *C. albicans* adhesins. We also found similar sequences in the other Als adhesins, usually at the same position near the N-terminus of the T domain [34]. There are also TANGO positive sequences in *S. cerevisiae* flocculins and other yeast adhesins (Figure 1; [35]). Like the Als sequences, they are rich in the *β*-branched aliphatic amino acids Ile, Val, and Thr. In contrast to the Als adhesins, other adhesins contain such amyloid-forming sequences within tandem repeats and so have multiple occurrences and can potentially form amyloids in several regions [34, 36].

**Figure 4:**
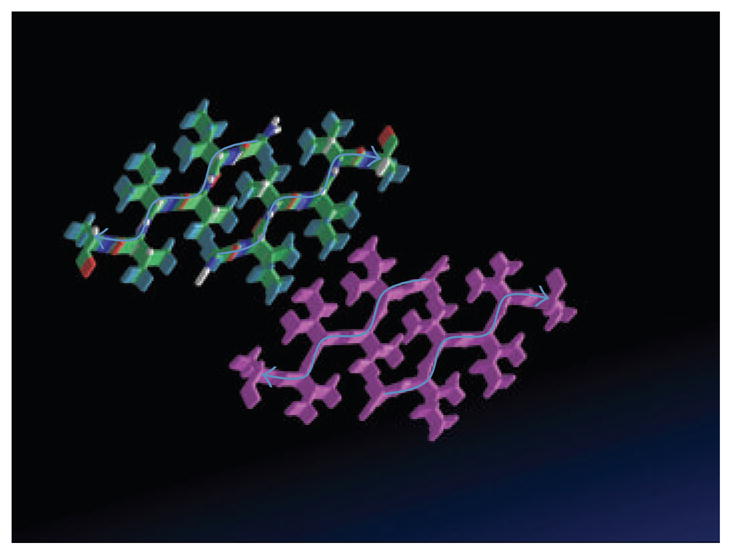
Model amyloid from the sequence GIVIVA from Als proteins. The individual peptides (each from one adhesin molecule) are stacked to form 4 *β*-sheets, each with 5 *β*-strands. The backbones of the peptides on top of each stack are marked with blue arrows. The sheets are paired through interactions of the hydrophobic side chains. Model was downloaded from Zipper DB http://services.mbi.ucla.edu/zipperdb/ [37].

Amyloid structures typically consist of 6–7 residue *β*-strands, with identical sequences from many molecules of the same protein assembled into *β*-sheets stabilized by energetically favorable interactions of the side chains. The *β*-strands are oriented perpendicular to the axis of the amyloid fiber, and the fiber axis lies in the plane of the *β*-sheet itself. Because the *β*-sheets are stabilized by close fit of the side chains, this structure is called a “steric zipper.” Typically, the most closely apposed residues interact anhydrously, with water being excluded from the intersheet region [37, 38]. Based on analysis of known structures and detailed modeling with energetics evaluation with ROSETTA, a web-based steric zipper geometric predictor is now available [37]. Steric zipper also shows positive predictions for the same sequence in Als proteins and in other Ile/Val/Thr rich sequences (Figure 4).

These predictions were borne out by *in vitro* testing. Synthetic peptides of the amyloid-predicted sequence shared by Als1p, Als3p, and Als5p formed amyloid fibers *in vitro*, as did large fragments of Als5p itself. Remarkably, the Als5p fragments, including an anchorless soluble version with 1351 amino acids, formed amyloid fibers at neutral pH at *μ*M concentrations [34, 35]. This is considerably lower than the predicted concentration *in vivo* in the immediate neighborhood of the cell surface, where the adhesin concentrations can be hundreds or thousands of times higher [10, 18, 39]. Similarly, TANGO-predicted peptides or protein fragments from unrelated adhesins also formed amyloid fibers under native-like conditions, including *C. albicans* Eap1 and the *S. cerevisiae* flocculins Flo1p and Flo11p [35]. Therefore, the amyloid predictions for the yeast adhesins were reflected in the solution properties of synthetic peptides and native proteins.

## 7. Two Approaches to Adhesin Amyloids

As we looked for consequences of amyloid formation *in vivo*, we were reminded of some unusual properties of Als-mediated adhesion. There were several properties reminiscent of amyloids. First, like amyloid formation, activation of adhesion and cell-cell aggregation was dependent on protein conformational change. Second, the conformational shift propagated around the entire cell surface and resulted in development of surface birefringence (light and dark surface areas observable when the sample is set between crossed polarizing filters). Such birefringence is a characteristic of local structural uniformity in subcellular-sized domains [40]. Third, the potentiation of adhesion was accompanied by exposure of hydrophobic regions on the cell surface, which we had assayed as binding of 8-anilino-1-naphthalene-sulfonic acid (ANS). Furthermore, high concentrations of ANS or Congo red inhibited the transition [25]. All of these phenomena were common between amyloid formation and Als5-mediated cell aggregation.

In the same year that we published these observations, Gaur and Klotz published a paper that characterized Als-mediated adhesion as a new kind of binding named “SRS”: stable, reversible, and specific [5]. Looking at the characteristics of whole cell binding to defined peptides displayed on magnetic beads, Gaur deduced that the Als5-mediated adhesion to beads was dependent on H-bonding between the adhesin and the peptide backbone of the ligand and was stabilized by side chain interactions. Conformational flexibility in the peptide was required for adhesion. In retrospect, this interpretation was a remarkable foreshadowing of the amyloid properties of the adhesins, because both types of interaction are critical for amyloid formation [27]. In fact, these properties might be needed for amyloid-dependent cell adhesion between adhesin molecules, or for ligand adhesin interactions, or for both.

We can now put these observations in the context of amyloid formation. H-bonding between backbone amides and carbonyl groups is the glue between the *β*-strands that make up each *β*-sheet. Side chain interactions are responsible for assembly of the sheets into amyloid fibers (Figure 4). Conformational flexibility in the polypeptide chains allows them to conform to the “templating” requirement to be added to the amyloid *β*-sheets. (The need for such conformational flexibility is also the reason that most proteins form amyloid only after denaturation, which exposes buried amyloid-forming sequences and allows them to adopt a configuration compatible with the *β*-sheet oriented perpendicular to the fiber axis.) In the fungal adhesins, the flexibility is an evolved property of the amyloid-forming regions of the proteins, so denaturation is not necessary for amyloid formation. We will revisit this idea later, when we discuss the sensitivity of the adhesins to extension forces and shear.

## 8. Amyloid Properties of Yeast Adhesins

These amyloid similarities were strongly supported by our analyses of the adhesion-activated state of cells expressing Als5p, as well as for some unrelated adhesins, the *S. cerevisiae* flocculins. Classic antiamyloid dyes such as Congo red, thioflavin S and thioflavin T stained the aggregates and, at high concentrations, inhibited aggregation activity. For flocculation, the amyloid perturbants were effective at 100- to 1000-fold lower concentrations than those used for carbohydrate competitive inhibitors of flocculation, which compete for binding to lectin sites in the N-terminal domains of the adhesins [35].

**Figure 5:**
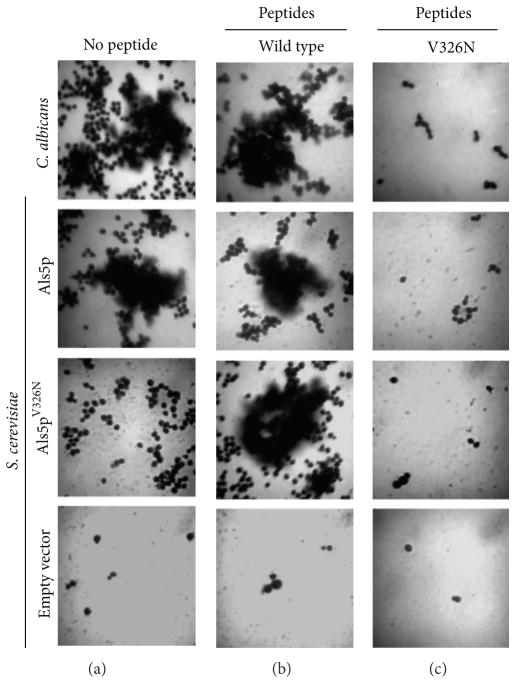
Yeast biofilms on polystyrene. 24-hour biofilms were grown from the cells listed on the left. Biofilms were grown in medium without (a) or in the presence of amyloid-forming peptide (b) or antiamyloid peptide (c). Reprinted with permission from Garcia et al. [41].

We also discovered a role for amyloids in formation of *S. cerevisiae* and *C. albicans* biofilms [41]. Expression of Flo adhesins or Als5p led to similar abilities to form biofilms on polystyrene, and amyloid-binding compounds could prevent biofilm formation (Figure 5). Thus, biofilm formation as well as fungal aggregation showed signs that amyloid formation was an essential step.

## 9. Effects of Sequence Change

We took a molecular genetic approach as well. Not surprisingly, the TANGO-positive sequence IVIVATT at position 325 in Als1p, Als3p, and Als5p has a strong propensity to form *β*-sheets. Several undergraduates worked to screen single site mutations in this sequence *in silico*, plugging many sequence variations into TANGO. We identified a potential mutated sequence, INIVATT, with *β*-aggregation potential in TANGO reduced 20- to 30-fold. This sequence retained a moderate potential to form *β*-strands. The substitution had the added advantages that it was charge-neutral, and the Asn residue has similar size and shape to the Val it replaces. A peptide with this mutated sequence did not form amyloid fibers [34].

We made this substitution, called V326N, in various versions of Als5. Soluble fragments of Als5p^V326N^ bound to fibronectin with affinity similar to the wild type versions, and the CD spectra were similar [41]. However, when the full-length Als5^V326N^ protein was expressed on the cell surface, the aggregation behavior was very different. When incubated with protein-coated beads in our aggregation assay, Als5p^V326N^ cells formed only small aggregates (3–5 cells per bead; compare to Figure 2). These small aggregates formed even in the presence of Congo red, thioflavin T, or thioflavin S, consistent with their residual activity being amyloid-independent [41]. Similarly, Als5p^V326N^ could not promote biofilm formation (Figure 5). This result confirmed that both activation of aggregation and biofilm formation depend on amyloid formation.

## 10. Effects of Homologous Peptides

We tested whether the nonamyloid V326N peptide might interfere with amyloid formation for Als5p. Indeed, at 200 *μ*g/mL (about 130 *μ*M) it effectively inhibited activation of cell adhesion, reduced the aggregates to a few cells, and inhibited biofilm formation [41]. Because the residual aggregates were amyloid-independent, they were not affected by Congo red. This is the result expected if the nonamyloid peptide binds to the amyloid and prevents further growth by blocking the interactions that would lead to growth of the amyloid structure. This activity would be analogous to the antiamyloid compounds that cap growing amyloids to block further growth of the *β*-sheets [42].

Conversely, the wild-type peptide rescued the cells expressing the mutant protein. Inclusion of this peptide at 2 *μ*g/mL (1.3 *μ*M) in aggregation assays led to formation of robust aggregates, which were disrupted with Congo red. These peptide activities are like transdominant mutations and are consistent with the exogenous peptide forming small aggregates and being able to serve as a template to force the V326N sequence into amyloid-like interactions. Therefore, the wild-type peptide has the ability to promote formation of amyloid interactions, and the nonamyloid peptide can disrupt these same interactions (Figure 5). These results further reinforced the idea that amyloid interactions are responsible for activation of cell aggregation and biofilm formation.

## 11. Single Molecule Studies and the Discovery of Surface Amyloid Nanodomains

Klotz recruited Yves Dufrêne, a world leader in use of atomic force microscopy (AFM), to determine properties of proteins. Dufrêne gave the problem to his student David Alsteens, who must have been destined to study his namesake proteins. He and Dufrêne discovered emergent properties of the Als molecules both *in vitro* and *in situ*. In fact, their observation of force-induced clustering was key to our realization that amyloid formation drives clustering of the adhesins on cell surfaces.

**Figure 6:**
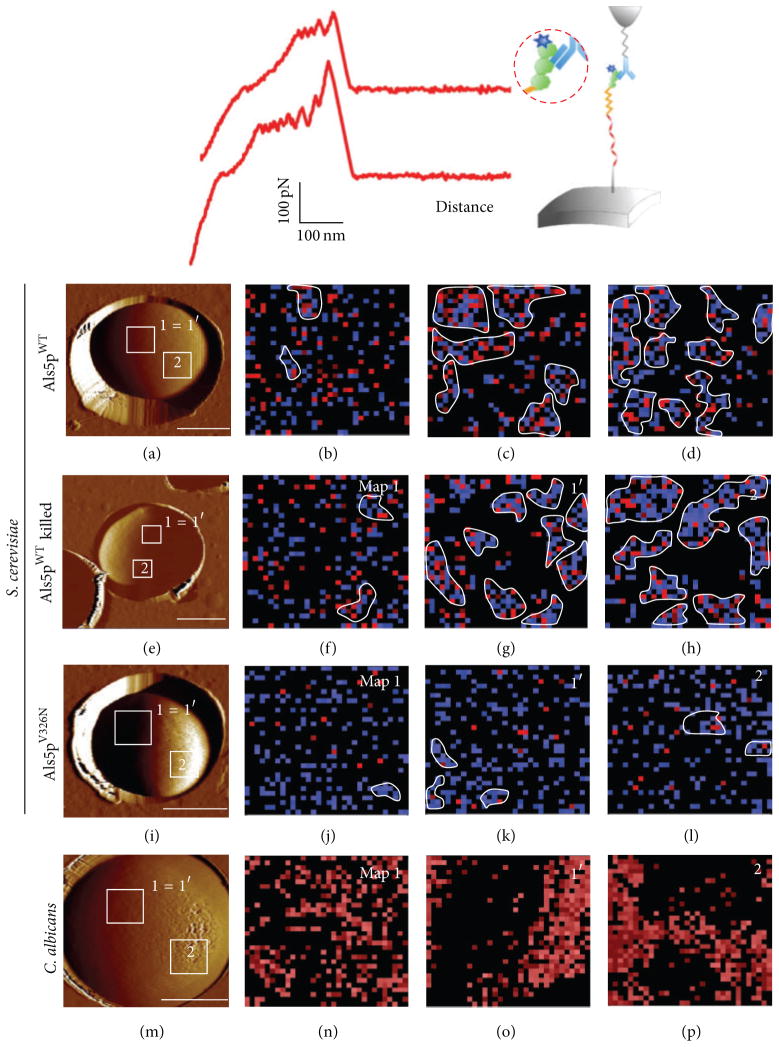
AFM experiments. Top: force-extension curve for unfolding of Als5p. From left to right, the T domain containing the amyloid sequence unfolds first, the TR domains (sawtooth pattern), and then the invasin Ig-like region. Below: maps of Als adhesins in 1 *μ*m^2^ areas of the surface of live cells and heat-killed cells. The first column shows an AFM image of the tested cell, with areas mapped marked as squares. The second column shows the first mapping (area 1); 2nd column shows a 2nd mapping of the same area (area 1′); 3rd column is mapping a remote area (area 2). Reprinted with permission from Alsteens et al. [45] and Garcia et al. [41].

AFM is complementary to molecular biology methods used to study Als adhesins in that it measures hydrophobicity, elasticity, extensibility, and binding energies of individual molecules at a defined location [43]. A macromolecule or cell is positioned on a planchet on the stage of the microscope and physically probed with a *μ*m scale “needle tip” made from a silicon nitride cantilever. In our studies, the tip is usually derivatized with a ligand for Als5p (often another Als5p molecule) or antibody to an epitope tag. When the tip is tethered to the planchet through its interactions with an adhesin, the tip deflects as it is retracted from the surface. The force applied is calculated, so we know how much resistance the adhesin molecule applies as it resists being stretched (Figure 6).

Our first studies looked at forces involved in unfolding the protein domains of Als5p N-terminal fragments (the Ig-like, T, and TR regions in Figure 1; [44]). When the tip pulled on an Als5p fragment with forces of 50–200 pN, the domains unfolded sequentially. Each unfolding event was accompanied by lengthening of the Als5p polypeptide. Furthermore, the unfolding order was reproducible. Based on the polypeptide lengths, we deduced that the T domains were the first to unfold, potentially exposing the amyloid sequences. Next, the tandem repeats unfolded one by one (Figure 6). The number of “teeth” in the unfolding curve corresponded exactly to the number of tandem repeats, with 6 in the Als5p allele that we have studied and up to 33 teeth in Als1p [45, 46]. Each tandem repeat added 8.4 nm to the length of the protein when it unfolded.

## 12. Mapping Yeast Surface Amyloids

The Dufrêne group then studied Als5p properties on the cell surface [44, 45]. Yeast cells embedded in a microporous membrane attached to the planchet presented cell surface Als molecules *in situ*. This configuration allowed us to monitor the unfolding of protein domains and also to map the distribution of Als adhesins on the surface of live *S. cerevisiae* or *C. albicans* cells. There was strong binding between the surface adhesins and tips derivatized with antibody to an N-terminal epitope tag or with tips derivatized with Als5p itself. Als proteins unfolded, as they had *in vitro*, except the molecules were extended to 200–300 nm before the AFM tip dissociated. The extra extension was due to the length of the C-terminal unstructured “tails.” Alsteens then “went fishing” with the probe as his fishing rod and mapped distribution of adhesins on the cell surface itself. The adhesins were randomly placed with surface densities of 153–172 adhesin proteins/*μ*m^2^. Surprisingly, casting again into the same surface area revealed a 1.5-fold increase in protein density and a 15-fold increase in clustered molecules; it was as if the “fish” had formed schools (Figure 6). The surfaces of heat-killed cells showed similar increases in adhesin density and clustering on the second time casting. In contrast, there was no clustering increase in cells expressing the nonamyloid mutant Als5p^V326N^. These adhesin clusters (“nanoadhesomes” or “nanodomains”) propagated around the cell surface, just like the propagation of adherence activation we had observed in 2004 [25, 45]. The obvious conclusion was that the nanodomains were composed of adhesion molecules aggregated through amyloid-like interactions right on the cell surface.

We fished with AFM tips derivatized with different “bait” molecules. Indeed, using our amyloid-forming 13-aminoacid peptide as bait showed an entirely new kind of interaction [47]. The peptide bound to clustered adhesin molecules, and retracting the tip led to a force-distance curve that looked like a zipper, with each tooth representing the rupture of a single H-bond. This result was similar to those found in probes of *β*-amyloid fibrils [48, 49]. Thus, Als5p can form amyloids with similar physical characteristics to the better known pathological amyloids.

The Dufrêne group has used the AFM to study effects of Als expression on surface properties for *C. albicans* during germ tube formation, which leads to a large increase in Als protein expression [46]. Concomitantly, there are increases in Als length (due to expression of more extended alleles), hydrophobicity of the cell surface, and mannoprotein expression and lengths. A similar remodeling of the cell surface of the germ tube follows treatment of cells with the antifungal glucan synthesis inhibitor caspofungin: there is increased Als expression and cell surface hydrophobicity [50]. In addition, there is a rougher cell surface, cell swelling, and a decrease in the mechanical cell wall strength.

Recently, Alsteens and A. Beaussart have attached intact cells to the AFM tip. These studies show the adhesive strength, extension, and elasticity of interactions between the cells, which are Als protein-dependent, and similar to those between individual Als molecules in the *in vitro* studies. Alsteens showed interactions between *C. albicans* cells and Als proteins attached to the planchet. Beaussart documented the adhesion between a *Staphylococcus epidermidis* cell attached to the AFM tip and a *C. albicans* cell on the planchet [51, 52]. Remarkably, germ tubes from *C. albicans* cells expressing Als1p and Als3p can bind to bacteria at strengths up to 9 nN and at tether lengths up to almost 1 *μ*m. So far, AFM experiments have shown the consistent binding interactions of substrates and Als, as peptide, soluble protein, and on the surfaces of yeast and fungal cells.

## 13. Fungal Catch Bonds

There is a remarkable behavior in lymphocytes and bacteria that adhere under flowing liquid: they bind more strongly under high shear than under low shear. This phenomenon is called catch bonding [53–56]. Recently, we have seen similar behavior in yeasts. Yeast cells that were vortex-mixed or sheared under laminar flow increased in adhesion and aggregation. Cells that were not vortex-mixed or under low shear flow did not increase in adhesion. The catch-bonding behavior in yeast is dependent on the formation of amyloids nanodomains on the cell surface. In the presence of force, the wild-type adhesins cluster into cell surface nanodomains, but our nonamyloid Als5p^V326N^ neither forms nanodomains nor increases adhesion in response to vortex mixing or laminar flow. As expected, amyloid-binding dyes thioflavin S and Congo red (*μ*M-mM) can prevent the increase in aggregation activated by force. The *S. cerevisiae* adhesins Flo1p and Flo11p/Muc1p showed similar amyloid-dependent catch bonding. So, when the yeast adheres to a substrate in the presence of flowing liquid, the adhesion is strong and resistant to shear-induced deadhesion. This adhesion leads to development of yeast biofilms. Thus, force sensitive formation of surface amyloid nanodomains is critical for formation of biofilms.

## 14. Amyloids in Host-Pathogen Interactions

Our *in vitro* studies convinced Klotz that there must be consequences for the presence of amyloids in host-microbe interactions. He arranged a luncheon in Tucson with Lipke and pathologist Richard Sobonya, who subsequently led investigations into presence of fungal amyloids in autopsy sections of invasive candidiasis victims [57]. We also utilized *Caenorhabditis elegans* as a model of infection and investigated the role of Als5p amyloids in host-microbe interactions [58]. Surprisingly, we found that in both systems Als5p amyloids function not in pathogenicity but rather in suppressing inflammation.

*C. elegans* is a microbivore nematode normally found in soil. It makes an excellent model organism for a variety of reasons: small size, ease of care, fast reproduction, large brood size, sequenced genome, real time observation of infectious process, and availability of mutants through the mail [59]. Consequently, *C. elegans* excels as a model organism to study host-microbe interactions, because many clinically important bacterial and fungal pathogens cause infections, and because monitoring lifespan is an easy assay for pathogenicity. Additionally, many components of the innate immune system are conserved between nematodes and humans. We determined the role that Als5p has in pathogenicity by feeding the nematode various yeast strains [58]. To our surprise, we found that *S. cerevisiae* was pathogenic, causing 50% death within about 60 hrs. Also surprisingly, expressing Als5p on the yeast cell wall delayed the mean time of death to 95 hours or more, the same as nonpathogenic *E. coli*. Additionally, the *C. elegans* fed Als5p-yeast retained yeasts in their intestines, while this was not the case for nematodes fed yeast not expressing the adhesin. These observations of normal lifespan with increased intestinal occupancy meet the criteria for commensalism, defined as a state of infection that results in either no damage or clinically unapparent damage to the host [60].

We hypothesized that the amyloid domain in Als5p might have a role in this commensal-like response. When we fed *S. cerevisiae* expressing Als5p with nonamyloid-forming substitution (V326N), the worms had increased mortality. The conclusion is that the amyloid domain of Als5p functions to promote commensalism, at least in nematodes.

**Figure 7:**
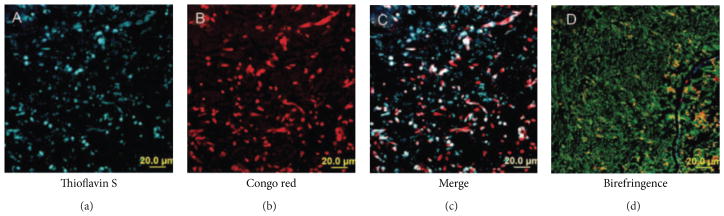
Amyloid staining of *C. albicans* cells in a GI tract abscess from a candidiasis victim. Reprinted with permission from Gilchrist et al. [57].

Are there amyloids on yeast surfaces in human infections? The answer is “Yes.” With Sobonya, pathologist Kevin Gilchrist surveyed autopsy tissues from gastrointestinal (GI) tracts of candidiasis victims, treating with antibodies to normal human amyloid proteins, but the results were all negative [57]. Nevertheless, M. Garcia, using confocal microscopy and thioflavin T and Congo red staining, demonstrated the stark beauty of *Candida* hyphae, pseudohyphae, and yeasts coursing through tissue from the human gastrointestinal tract (Figure 7). We also encountered an entirely novel participant in the pathogenesis of invasive candidiasis, namely, serum amyloid P component (SAP). Gilchrist found that the fungi were coated with this antigen, and Garcia showed that *in vitro* SAP binds to *C. albicans* or to Als5p surface amyloids on *S. cerevisiae* [57]. We have recently extended our observations to show that cell surface functional fungal amyloid and SAP deposition occur in other fungal diseases in humans, specifically invasive *Aspergillus*, *Coccidioides*, and *Mucorales*. SAP has anti-inflammatory activity and therefore may prevent massive inflammatory responses and ensuing tissue damage in the presence of a “normal” amyloid load [61]. Presence of SAP in fungal abscesses was correlated with a lack of inflammatory infiltrate.

Therefore, our studies of host-pathogen interactions in worms and humans lead us to similar conclusions. In both hosts, cell wall amyloids promote a noninflammatory, commensal-like state.

## 15. Some Questions Not yet Answered

There are obvious questions that need to be answered as well as some foreseeable practical applications of the new knowledge of functional amyloids in cell adhesion. So we will ask questions first and then predict a few research areas.

A first question is whether functional amyloids cluster any cell adhesion molecules in other fungi, or in clades other than fungi. There are indeed TANGO-predicted amyloid sequences in many other fungal adhesins, in bacterial adhesins, and in mammalian cell adhesion molecules [35, 39], but these predictions show only potential, not amyloid formation or function in adhesion. The demonstrated presence of functional amyloids in *S. cerevisiae* Flo proteins as well as on the surface of pathogenic *Aspergillus*, *Coccidioides*, and *Mucorales* argues that the phenomenon is widespread among the fungi.

There is excellent evidence for amyloid function in activity of bacterial adhesins. Functional amyloid interactions mediate assembly of adhesive appendages called curli in gram-negative bacteria. A complex network of chaperones shepherds the amyloid-forming proteins through the secretion pathway and mediates assembly as the subunits pass through the outer membrane [62]. Mature curli mediate substrate adhesion and biofilm formation. Hydrophobic chaplins of *Streptomyces coelicolor* assemble through amyloid interactions to mediate formation of biofilm-like pellicles at air-water interfaces [63, 64]. In gram positives, an amyloid-forming domain is key to activity of *Streptococcus mutans* P1 adhesin and probably other adhesins as well. Therefore, oral biofilms show amyloid properties, as do environmental biofilms [65–67]. Amyloid interactions are also present in *Bacillus subtilis* biofilm matrices [68].

By analogy with bacterial systems, we can anticipate several areas of research. First, secretion and anchorage of the fungal adhesins should require an extensive and perhaps novel chaperone network in the ER, Golgi, and secretory vesicles. The chaperones must prevent premature assembly of the amyloids during passage through the secretory pathway and may also mediate cross-linking to the cell wall glucan matrix [12].

Metazoan cell adhesion molecules have some domains homologous to fungal adhesins, and they contain predicted amyloid-forming sequences, including some that are rich in Ile, Val, and Thr [39, 69]. However, we have no experimental evidence to back up these bioinformatic inferences. A first step might be to assay for inhibition of cell adhesion by amyloid perturbants like Congo red, thioflavin T, or thioflavin S.

As another area of research, we have uncovered unexpected consequences of surface amyloids in host responses to infection. We have shown initial responses in innate immunity in humans and in *C. elegans*. It remains to be seen whether immune responses to fungal amyloids are uniformly anti-inflammatory, or if the idea is a result of having only the first two examples. Clearly, there is great discriminatory power in the general approach of challenging immune cells or host organisms with model fungi that express amyloid forms of surface proteins, then comparing the responses to proteins with mutations in the amyloid sequences. This approach represents an opportunity to explore the ramifications of immune response to a specific class of structures in pathogenic organisms.

The influence of functional amyloids in cell-cell, cell surface interaction and biofilm formation and persistence will continue to evolve, as more and more focus is placed on the role these phenomena play in microbial pathogenesis and environmental contamination. In the clinic, persistent bacterial and fungal infections are increasingly becoming more costly and difficult to treat, as many microbes acquire resistance to first-line drugs. In this setting, one could envision targeting amyloid interactions to reduce pathogen-host cell adhesion in combating persistent infections. This approach has the advantage of reducing the reliance on current antifungals and other antibiotics, and thereby the emergence of resistance. Combination of antiamyloid dispersion methods with lower doses of antimicrobial drugs could conceivably affect the removal of dormant persister cells hidden within a biofilm matrix. Similar research is currently being actively pursued for bacterial biofilms [62, 70–72].

Perhaps, based on knowledge of the degree of conservation present in fungal and bacterial amyloid forming sequences, peptides, antibodies, and small molecules can be used to develop rapid detection methods. These findings could then be used to inform treatment and eradication of persistent infection.

## 16. Summary

So a series of meetings and collaborations, often helped by lunch dates, led to interactions between scientists with diverse approaches to the problem of fungal pathogenesis. Meetings between a biochemist, a medical mycologist, a biophysicist, and a pathologist led to discovery of fungal adhesin amyloids. Fungal adhesin amyloids now have demonstrated roles in cell adhesion, biofilm formation, and host response. The consequences of the discovery are only beginning to be known, and their range of consequences is still expanding fast. So the moral of the story is the next time someone says, “Let’s do lunch,” take them seriously indeed.
